# Interleukin-4 Ameliorates the Functional Recovery of Intracerebral Hemorrhage Through the Alternative Activation of Microglia/Macrophage

**DOI:** 10.3389/fnins.2016.00061

**Published:** 2016-03-08

**Authors:** Jianjing Yang, Saidan Ding, Weilong Huang, Jiangnan Hu, Shengwei Huang, Yu Zhang, Qichuan Zhuge

**Affiliations:** ^1^Department of Neurosurgery, First Affiliated Hospital of Wenzhou Medical UniversityWenzhou, China; ^2^Zhejiang Provincial Key Laboratory of Aging and Neurological Disease, Wenzhou Medical UniversityWenzhou, China

**Keywords:** stroke, intracerebral hemorrhage, interleukin-4(IL-4), microglia/macrophage, recovery, M1 phenotype, M2 phenotype

## Abstract

Neuro-inflammation plays an important role in the recovery of brain injury after stroke. Microglia/macrophage is the major executor in the neuro-inflammation, which can be polarized into two distinct phenotypes: injurious/toxic classical activation (M1 phenotype) and protective alternative activation (M2 phenotype). Here, we investigated whether intracerebral administration of interleukin-4 (IL-4) at an early stage could affect the activation of microglia/macrophage and the corresponding outcome after intracerebral hemorrhage (ICH). The neuro-behavior was recorded between different groups in the rat ICH model. The M1 and M2 markers were then determined by qRT-PCR, western blotting, ELISA, and immunofluorescence, respectively. We observed aberrant activation of microglia/macrophage after ICH. After intracerebral injection of IL-4, M1 activation was greatly inhibited while M2 activation was enhanced, along with improving neurobehavioral recovery from deficits after ICH. Our study showed that early intracerebral injection of IL-4 potentially promotes neuro-functional recovery, probably through enhancing the alternative activation of microglia/macrophage.

## Introduction

Intracerebral hemorrhage (ICH) accounts for 15% of strokes, which is a lethal neurological disease (Qureshi et al., 2001; Mehndiratta et al., 2012). Treatment with ICH generally involves a surgical procedure to remove the hematoma, with varying and controversial efficiencies and prognosis. Due to the poor prognosis of ICH coupled with the extensive burden on the family of patients and society, exploring new ways to improve the clinical outcome of ICH is necessary. Recent studies showed that neuro-inflammation deeply relates to the progression of ICH-induced brain injury (Wang and Tsirka, 2005; Mracsko and Veltkamp, 2014). Progressive brain loss after stroke could be prevented and the clinical outcome could be improved by modulating the immune milieu after ICH (Wang et al., 2007).

Microglia/Macrophage plays a critical role in innate immune in CNS, which has two cell types: injurious/toxic classically activated microglia/macrophage (M1) and protective alternatively activated microglia/macrophage (M2) (Gordon, 2003; Martinez et al., 2008; Kigerl et al., 2009; Tang et al., 2014). Classically activated cells could produce reactive oxygen species (ROS) and pro-inflammatory cytokines which are cytotoxic, while alternatively activated cells could release anti-inflammatory cytokines and neurotrophic factors, which show the characteristics of wound-healing by blocking pro-inflammatory response (Banati et al., 1993; Kigerl et al., 2009). These two activating statuses have opposite roles during the repair after CNS damage. Manipulating the activation status of the microglia/macrophage may be a promising strategy to improve the outcome after ICH.

Recently, Interleukin-4 (IL-4) cytokines has been demonstrated to effectively induce protective M2 cell subtype activation, which associated with tissue repair and composition of extracellular matrix (Gordon, 2003; Colton and Wilcock, 2010; Graeber, 2010). Also, pretreatment by IL-4, prevented, in a dose-dependent manner, neuronal cell injury induced by activated microglia (Chao et al., 1993). Here, in the rat ICH model, we focus on whether IL-4 could improve the outcome by modulation of inflammatory milieu and its influence on microglia/macrophage polarization after administration of IL-4.

## Materials and methods

### Animal groups and ICH model

A total of 167 adult male Sprague Dawley (SD) rats that weighed 250–300 g were randomly allocated to four groups: Control group (*n* = 12), ICH group (*n* = 73), ICH + PBS group (vehicle group, *n* = 41), and ICH + IL-4 group (*n* = 41). In detail, each time point has six rats in qRT-PCR experiment. Each group has eight rats in behavior recovery test experiment and each group has three rats in western blotting, ELISA, and immunofluorescence. The ICH model was induced by intra-striatal injection of collagenase IV as previously described (Lu et al., 2014; Chen M. et al., 2015). Briefly, we performed rat anesthesia with intraperitoneal injection of 10% chloralhydrate (0.4 ml/kg) which diluted in water. Then, the rats were fixed with stereotaxic frame (KOPF, California, USA), the bregma exposed and drilled a pin hole located on right lateral 3 mm away from bregma. The needle was inserted into striatum (5 mm depth from skull pin hole) controlled with stereotaxic frame. One microliter collagenase IV (0.25 IU/μl) (Sigma; C5138) was slowly injected, which was controlled by micro-injector lasting for 5 min. Animal Care Committee of the Wenzhou Medical University approved the procedures involving animals. All surgeries were performed under chloralhydrate anesthesia, for minimizing suffering.

### Drug injection

The IL-4 (PeproTech Asia, # 400-04) was dissolved in PBS and stored at −20°C. IL-4 (0.8 μl each, final concentration is 0.25 μg/μl) was orthotropic intracranially injected 1 h post collagenase IV injection. The injection of IL-4 was controlled by stereotaxic frame and micro-injector. The injection coordinate is the same as that of collagenase IV injection. For control, the ICH+PBS group (vehicle group) has been injected with same volume of PBS (0.8 μl).

### Gene expression

The striatum were separated from rat brains and the total mRNA was then isolated from striatum with Trizol (Thermo Fisher, 15596-026). RevertAid TM First Strand cDNA Synthesis Kit (Thermo Fisher, K1622) was used to reverse transcribe 1 μg of total mRNA to cDNA. Then, SYBR Green Realtime PCR Master Mix-Plus (TOYOBO, QPK 212) was used to perform qRT-PCR. Primers used in qRT-PCR reactions were synthesized by Invitrogen (Shanghai, China) (Table 1). The fold change of gene expression was calculated using the 2^−ΔΔCt^ algorithm.

**Table 1:** **Primers used in qRT-PCR reactions**.

**Gene**	**Forward primer(5′-3′)**	**Reverse primer (5′-3′)**
iNOS	AAGCTGCATGTGACTCCATC	TGCAAGAGATATCCGAGGTG
TNF-α	GCGTGTTCATCCGTTCTCTACC	TACTTCAGCGTCTCGTGTGTTTCT
IL-1β	TACAAGGAGAGACAAGCAACGACA	TTCCATCTTCTTCTTTGGGTATTG
IL-13	CCACAGGACCCAGAGGATATTGA	TAGCGGAAAAGTTGCTTGGAGTAA
IL-6	AGACTTCACAGAGGATACCACCCAC	CAATCAGAATTGCCATTGCACAA
Arg 1	CATATCTGCCAAGGACATCG	ATTCCCAGCTTGTCCACTTC
Ym 1	GGAGTAGAGACCATGGCACTGAAC	GACTTGCGTGACTATGAAGCATTG
CD206	TCTTTGCCTTTCCCAGTCTCC	TGACACCCAGCGGAATTTC
GAPDH	CTGGCATTGCTCTCAATGACAAC	CTTGCTCTCAGTATCCTTGCTG

### Western blot analysis and enzyme-linked immunosorbent assay (ELISA)

Before the surgery, the rats were anesthetized with 10% chloralhydrate. Then, we separated striatum from rat brains on ice. Each striatum was homogenized in RIPA lysis buffer (Thermo, USA) with the addition of protease inhibitor. The homogenate was centrifuged for 15 min at 12,000 rpm, 4°C. The protein concentrations were detected by Pierce™ BCA Protein Assay Kit (Thermo Scientific, # 23225). Fifty micrograms of total proteins were loaded onto 8% SDS-PAGE gel. Proteins were transferred onto polyvinylidene difluoride filters (PVDF) membranes, followed by blocking with 5% milk for 1 h at room temperature. Then, specific primary antibodies against iNOS (Rabbit; Abcam; ab3523; 1:3, 000); Arg 1 (Rabbit; Abcam; ab91279; 1:1, 000); and tubulin β (Rabbit; Bioworld Technology; BS1482; 1:1, 000) were incubated overnight at 4°C. After the incubation with secondary antibodies for 1 h, we used ECL (Thermo Scientific; PI32109) to visualize bands, which were then analyzed by the Quantity One (Bio-Rad; Version 4.6.2).

Also, we used ELISA kits (R&D Systems, USA) to detect the cytokines in the striatal protein extract including IL-1β, TNF-α, IL-6, and IL-13, according to the manual instruction.

### Immunofluorescence

The rats were anesthetized at day 3 after ICH. Trans-cardiac perfusion has been performed with 4% paraformaldehyde, followed by dehydration with 30% sucrose for 48 h. Coronal frozen sections (10 μm) were then harvested from the striatum. Brain sections were incubated overnight with primary antibodies: iNOS (Rabbit; Abcam; ab3523; 1:100); Arg 1 (Rabbit; Abcam; ab91279; 1:100); and Iba 1 (Goat; Abcam; ab5076; 1:200) at 4°C. Then, the slices were incubated at 37°C for 1 h with secondary antibodies: Fluor 594-conjugated (Donkey anti-Rabbit; Invitrogen; A-21207; 1: 500) and Fluor 488-conjugated (Donkey anti-Goat; Invitrogen; A-11058; 1: 500). Furthermore, sections were stained with DAPI (Roche; 10236276001; 1:20, 000). Images were acquired by using scanning fluorescence microscope (Leica Microsystems). For statistical analysis, we selected 6 slices for staining from each brain (*n* = 3 for each group). Six to ten digital microscopic images around the hematoma region were randomly applied in each slice. Double positive cell number was quantified and the average of sections from each brain was taken. The size of the image was measured by scanning fluorescence microscope. Results were expressed as the average number of cells per mm^2^.

### Behavior examination

The assessment of the modified Neurological Severity Scores (mNSS) including sensory, motor, reflex, and balance tests is a complex behavior test (Schallert et al., 1997; Mahmood et al., 2013). The maximum score of the test is 18, which means maximum deficit. The rats had been trained and assessed prior to surgery, to make sure the basal score is 0. Then, blind behavioral deficit tests were performed at 1, 3, 7, and 14 days after ICH, respectively.

### Statistical analysis

Data were presented as mean ± SEM. Two-way analysis of variance (ANOVA) and Bonferroni post-tests were used to analyze the behavior data. The gene expression, western blotting, ELISA, and immunofluorescence data were analyzed by One-way ANOVA and Bonferroni post-tests. The results were considered statistically significant when *P*-value < 0.05. The statistical analysis was performed by GraphPad Prism 5 (San Diego, CA, USA).

## Results

### The M1/M2 cell subtype activation during the acute inflammation after ICH

**Figure 1:**
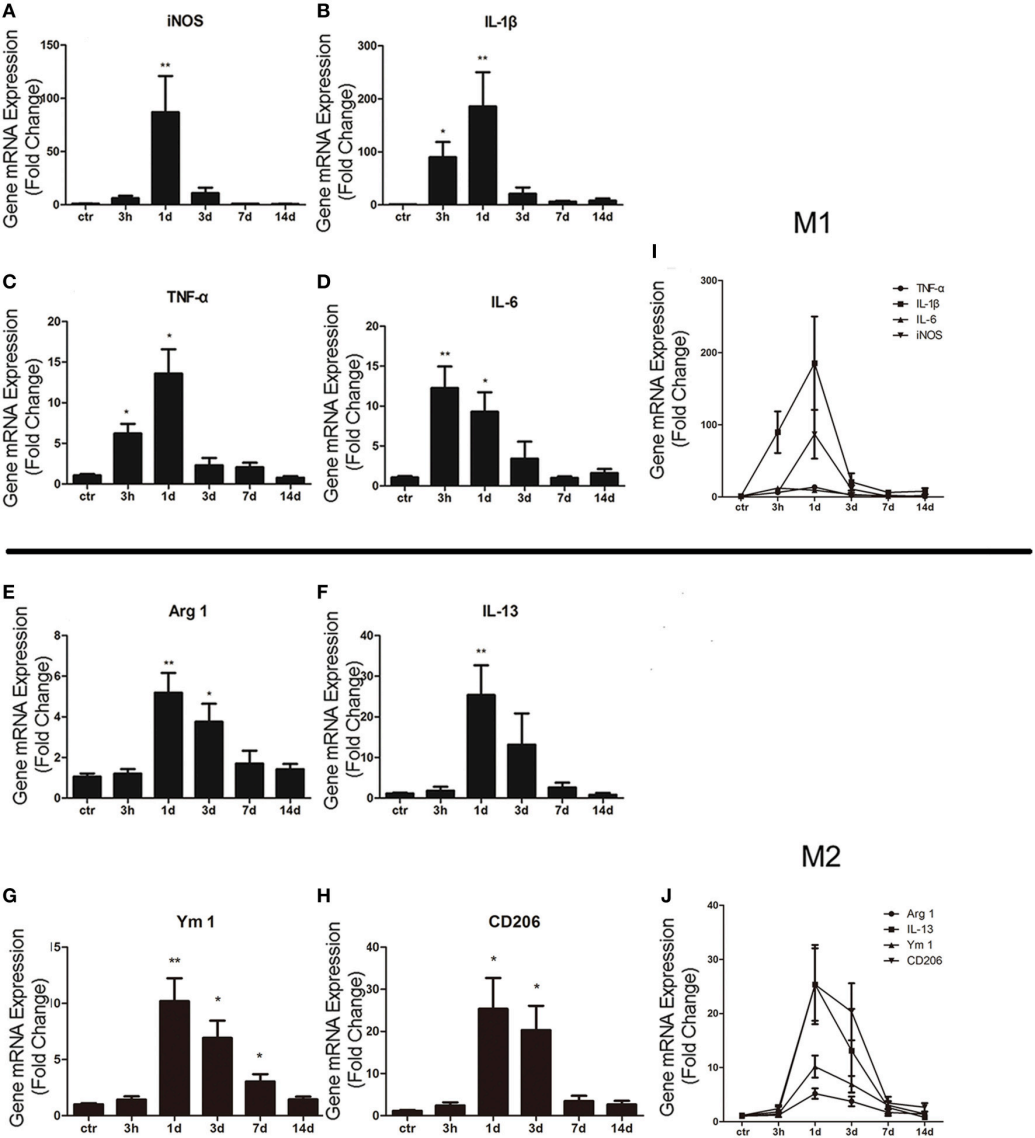
**M1 and M2 microglia/macrophage activation in the striatum after intracerebral hemorrhage (ICH)**. Real-time RT-PCR results showed time-course changes in M1 activation-related genes **(A–D,I)**. qRT-PCR results showed time-course changes in M2 activation-related genes **(E–H,J)**. ^*^P < 0.05; ^**^P < 0.01 (*n* = 6).

In order to study the activation of M1/M2 cells after ICH, total mRNA was isolated and purified from striatum. qRT-PCR was then performed to respectively test M1 biomarkers (such as iNOS, IL-1β, TNF-α, and IL-6) and M2 biomarkers (such as IL-13, Arg 1, Ym 1, and CD206). For M1 makers, we found that the expression level of iNOS (*P* < 0.01; *n* = 6) was significantly increased at 1 day post ICH (Figure 1A), while those of IL-1β (*P* < 0.05; *n* = 6), TNF-α (*P* < 0.05; *n* = 6), and IL-6 (*P* < 0.01; *n* = 6) were increased at 3 h post ICH (Figures 1B–D). For M2 markers, the levels of Arg 1 (*P* < 0.01; *n* = 6), IL-13 (*P* < 0.01; *n* = 6), YM 1 (*P* < 0.01; *n* = 6), and CD206 (*P* < 0.05; *n* = 6) were all increased at 1 day post ICH (Figures 1E–H). The results showed that the M1 activation was rapidly increased at 3 h, peaked at 1 day, and quenched to the normal about 3 days after ICH (Figure 1I). Whereas, the M2 activation was increased at 1 day, which was later than M1 activation, and returned after about 7 days (Figure 1J). In summary, after ICH, both M1 and M2 subtypes are significant activated, while M2 activation is occurred later than M1, but persisted for a long time.

### IL-4 improved the function recovery of ICH rat

**Figure 2:**
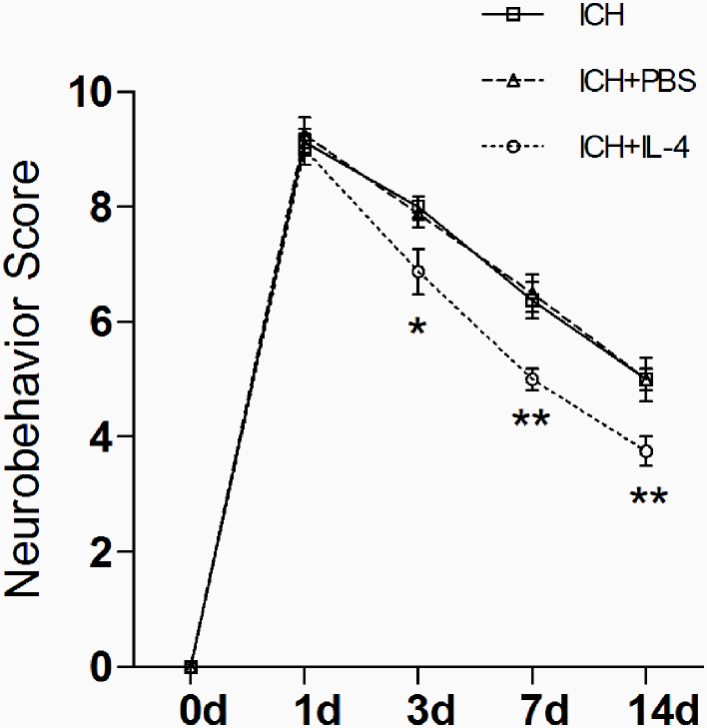
**IL-4 improves the recovery of neurobehavioral function after ICH**. The neurobehavior was evaluated by mNSS after ICH between different groups. ^*^P < 0.05; ^**^P < 0.01 (*n* = 8).

Then, we studied whether the administration of IL-4 could influence neurobehavioral function recovery in ICH model rats. We used the mNSS scale to evaluate neurobehavioral function between different groups. The rats were trained prior to surgery to ensure the basal level (score = 0). Then, the rat behavior score was evaluated and record at each time point post ICH. The evaluation process was blinded. We performed two-way analysis of variance (ANOVA) and Bonferroni post-tests to analyze the result of the behavior test. We found that there were significant differences between the ICH+IL-4 group and the ICH+PBS group at 3 (*P* < 0.05; *n* = 8), 7 (*P* < 0.01; *n* = 8), and 14 days (*P* < 0.01; *n* = 8) after ICH (Figure 2). Moreover, there was no significant difference between the ICH+PBS group and the ICH group (Figure 2). The results suggested that injection of IL-4 significantly improved the outcome of ICH model rats.

### IL-4 decreased the activation of M1 cells after ICH

**Figure 3:**
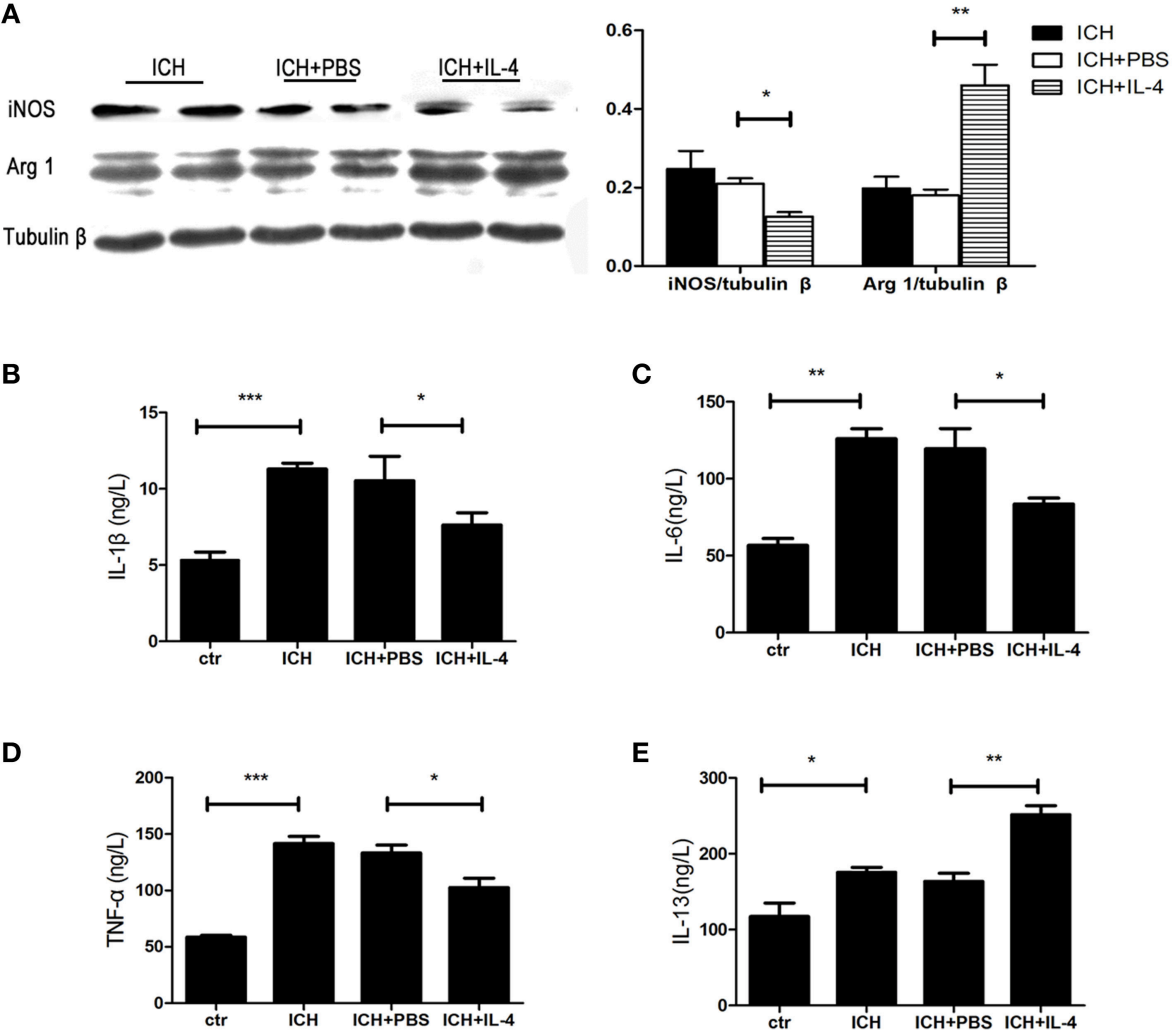
**IL-4 inhibits M1 activation and promotes M2 activation**. The protein level of M1 and M2 markers has been detected by western blot **(A)** and ELISA **(B–E)** at 1 day after ICH. ^*^P < 0.05; ^**^P < 0.01; ^***^P < 0.001 (*n* = 3).

**Figure 4:**
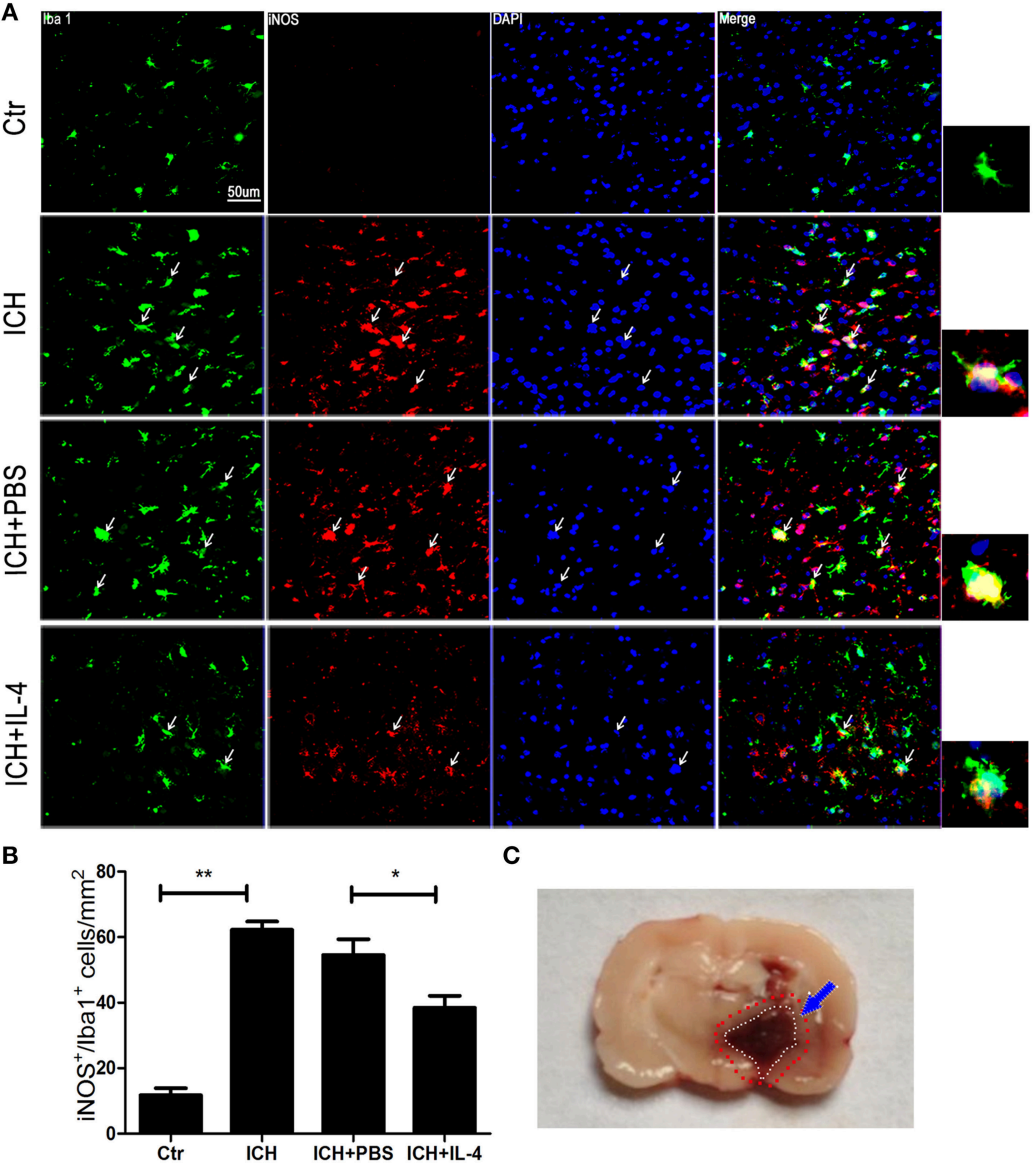
**IL-4 decreased iNOS+/Iba 1+ cells**. Compared with ICH group, iNOS+/Iba 1+ **(A,B)** cells had significant decreased in the ICH+IL-4 group at at 3 days after ICH. Arrows indicate iNOS+/Iba 1+ cells. Rat ICH model brain coronal section slice **(C)**. The region between the red and white cycle indicates the area around hematoma, where take images. Scale bar = 50 μm. ^*^P < 0.05; ^**^P < 0.01 (*n* = 3).

After we observed that IL-4 injection significantly improved the functional recovery, we explored the possible mechanism of IL-4 influences on the outcome of ICH. We performed western blot and ELISA to detect the expression levels of M1 markers. The results showed that iNOS (*P* < 0.05; *n* = 3), IL-1β (*P* < 0.05; *n* = 3), IL-6 (*P* < 0.05; *n* = 3), and TNF α (*P* < 0.05; *n* = 3) were downregulated at 1 day after administration of IL-4 (Figures 3A–D), which were compared to the ICH+PBS group. Furthermore, at tissue level, we co-stained brain slices with iNOS/Iba 1. We found that IL-4 could significantly decrease the iNOS^+^/Iba 1^+^ cells (*P* < 0.05; *n* = 3) surrounding the hematoma at 3 days after ICH (Figure 4). All above, the results indicated that the activation of M1 cells has been inhibited after IL-4 administration.

### IL-4 increased the activation of M2 cells after ICH

**Figure 5:**
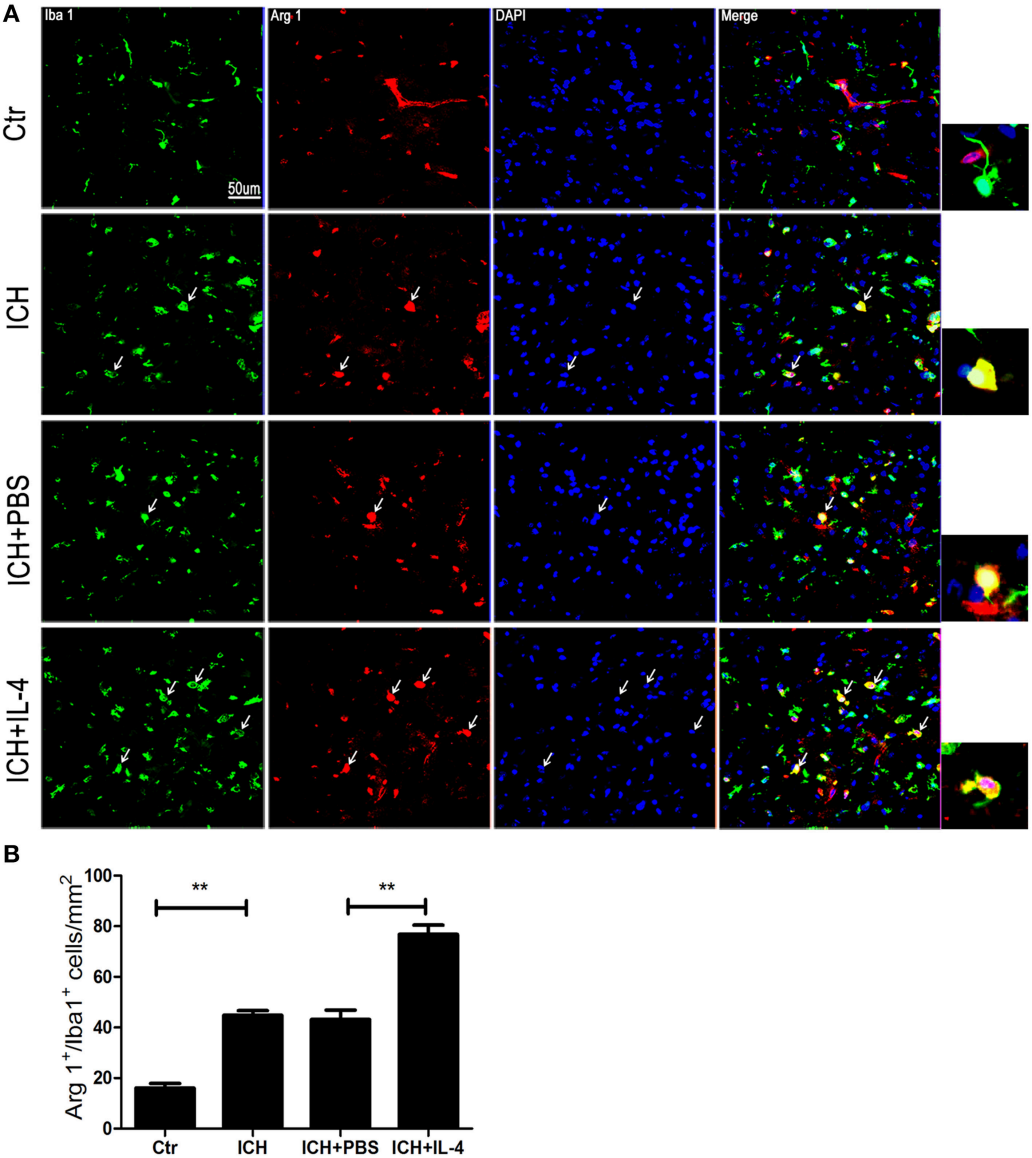
**IL-4 increased Arg 1+/Iba 1+ cells**. Compared with ICH group, Arg 1+/Iba 1+ **(A,B)** cells had significant increased at the ICH+IL-4 group at at 3 days after ICH. Arrows indicate Arg 1+/Iba 1+ cells. Scale bar = 50 μm. ^**^P < 0.01 (*n* = 3).

On the other hand, we also explored whether IL-4 influenced the activation of M2 cells. The M2 activation post IL-4 injection was investigated with western blot, ELISA and immunofluorescence, respectively. We found that IL-4 significantly induced Arg 1 expression (*P* < 0.01; *n* = 3) at 1 day after ICH (Figure 3A). Besides, ELISA results showed that injection of IL-4 enhanced the production of IL-13 (*P* < 0.01; *n* = 3) at 1 day after ICH (Figure 3E). In addition, at tissue level, the co-staining Arg 1/Iba 1 showed that Arg 1^+^/Iba 1^+^ cells (*P* < 0.01; *n* = 3) had been significantly increased in the ICH+IL-4 group (Figure 5) compare to ICH+PBS group at 3 days after ICH. These results demonstrated that IL-4 treatment could enhance the activation of M2 cell type after ICH.

## Discussion

In this study, we observed the dynamic activation of microglia/macrophage during the acute inflammation after ICH. Early intracerebral injection of IL-4 could improve the recovery of ICH rat. In addition, administration of IL-4 could change the polarization of microglia/macrophage by inhibiting M1 activation and inducing M2 activation.

Recent studies demonstrated that immune responses play an important role in the brains injury after stroke (Dirnagl et al., 1999; Xie et al., 2014; Yang et al., 2015). For innate immune, when encountering insulting stimulations, microglia/macrophage was activated (Zhang et al., 2001). Consistently, our study showed that the microglia/macrophage was significantly activated after ICH. Specifically, we found that the expression levels of M1 markers (IL-1β, TNF-α, and IL-6; Mosser and Edwards, 2008; Kumar et al., 2013; Tang and Le, 2016) rapidly increased at 3 h, peaked at 1 day, and returned to normal levels after about 3 days, whereas those of M2 markers (Arg 1, IL-13, YM 1, and CD206) (Kumar et al., 2013; Mosser and Edwards, 2008; Tang and Le, 2016) increased at 1 day after ICH and returned after about 7 days. The profile of M1/M2 changes is similar with that of the cutaneous wound model, performed by Deonarine et al. (2007) They found that both of M1 and M2 markers increased at day 2, with M2 markers increased consistently at day 4 and 8 during the healing of cutaneous wound (Deonarine et al., 2007). In addition, recent studies demonstrated that microglia/macrophage activation was detected as early as 1 h following ICH in the collagenase injection model and within 4 h using whole blood injection (Xue and Del, 2000; Wang and Doré, 2007). In spinal cord injury, traumatic brain injury and ischemic injury, M2 type transiently detected at high levels before 7 days, however, M1 type maintained even up to 28 dpi (Hu et al., 2012; Chen Y. J. et al., 2015; Kumar et al., 2015). The profile of microglia/macrophage activation was also positively associated with the severity of the model.

Evidence showed that IL-4 plays critical roles in learning and memory in the normal brain (Derecki et al., 2010). In addition, IL-4 has been demonstrated to protect the brain from injury in the ischemic model (Xiong et al., 2011; Hu et al., 2015). Administration of IL-4 at the acute phase of ICH in this study could significantly improve neuro-behavior recovery in ICH rat model. However, further studies are needed to test the protective effects of IL-4 for fully elucidate the optimal time point and dose.

Interleukin-4 (IL-4) is a multifunctional cytokine, which could influence Th2 subset differentiation and polarization of microglia/macrophage (Kato and Nariuchi, 2000; Kobayashi et al., 2013; Orihuela et al., 2016). Recent research points to a dual role of microglia/macrophage in neuronal injury and recovery in stroke. In addition, microglia/macrophage own two phenotypes (M1/M2), which plays opposite roles during the repair after CNS damage (Kigerl et al., 2009). The M2 type is related to wound healing and tissue repair can secret protective cytokines, while the M1 type involves in the pro-inflammation response exacerbate neuronal demise (Banati et al., 1993). Furthermore, the M1 type cells have been shown to impair axon regrowth (Kigerl et al., 2009), while M2 polarization has been shown to be essential for efficient remyelination (Miron et al., 2013). In the present research, administration of IL-4 at early stage after ICH decreased M1 activation, while enhanced M2 activation. Besides, IL-4 inhibited the secretion of M1-related cytokines (IL-6, IL-1β, and TNF α), while promoted the production of M2-related cytokines (IL-13). Thus, IL-4 modulate immune responses after ICH through changing the activation phenotypes of microglia/macrophage, which may partially account for the recovery of ICH rat by IL-4 administration.

However, for clinical application, there are still some issues need to figure out. The recombinant proteins still have some limitations as therapeutic agents (Leader et al., 2008). Also, the therapeutic window and the delivery method of the IL-4 still need to be further studied.

In conclusion, our study showed that microglia/macrophage was activated after ICH. Administration of IL-4 could improve the functional recovery of ICH. Early injection of IL-4 could promote anti-inflammatory response which favors the repair via inhibiting M1 cell activation while enhance M2 cell activation after ICH. Therefore, administration of IL-4 in the early phase of ICH is likely to be one promising therapeutic approach for improve the outcome of ICH.

## Author contributions

The manuscript has been approved by all authors. JY designed and performed the major study, analyzed data, and wrote the manuscript. SD and WH also performed part of research and data analysis. JH and SH helped the revision of this research. QZ and YZ designed the study and obtained funding for the study.

### Conflict of interest statement

The authors declare that the research was conducted in the absence of any commercial or financial relationships that could be construed as a potential conflict of interest.
